# Management of Contaminated Autologous Grafts in Plastic Surgery

**Published:** 2008-04-22

**Authors:** Robert F Centeno, Ankit R Desai, Marla E Watson

**Affiliations:** Saint Croix Plastic Surgery & MediSpa, Christiansted, US Virgin Islands 00824; Division of Plastic and Reconstructive Surgery, St Louis University, St Louis, Missouri; Body Aesthetic Plastic Surgery & Skincare Center, St Louis, Missouri

## Abstract

**Background:** Contamination of autologous grafts unfortunately occurs in plastic surgery, but the literature provides no guidance for management of such incidents. **Methods:** American Society of Aesthetic Plastic Surgery members were asked to complete an online survey that asked about the number and causes of graft contaminations experienced, how surgeons dealt with the problem, the clinical outcomes, and patient disclosure. **Results:** Nineteen hundred surgeons were asked to participate in the survey, and 223 responded. Of these, 70% had experienced at least 1 graft contamination incident, with 26% experiencing 4 or more. The most frequently reported reason for graft contamination was a graft falling on the floor (reported by 75%). Nearly two thirds of the contaminated grafts related to craniofacial procedures. Ninety-four percent of grafts were managed with decontamination and completion of the operation. The most common method of decontamination was washing with povidone-iodine, but this practice is contrary to recommendations in the literature. Only 3 surgeons (1.9%) said a clinical infection developed following decontaminated graft use. Patients were not informed in 60% of graft contamination incidents. The survey results and review of the literature led to development of algorithms for the management of inadvertent graft contamination and patient disclosure. **Conclusions:** Although autologous grafts do become contaminated in plastic surgery, the overwhelming majority can be safely decontaminated and produce minimal or no clinical sequelae. The algorithms presented are intended to serve as guides for prevention of contamination events or for their management should they occur.

The use of autologous grafts in aesthetic and reconstructive surgery has become increasingly common, especially with the growing popularity of fat and cartilage autografts in facial and nasal surgery. However, as with any technique innovation, the benefits come with some potential risks. Since contaminated grafts have the potential to grow bacteria, patients who receive them are theoretically placed at increased risk for infection.

This study was designed to survey plastic surgeons on their practices following inadvertent contamination of autografts. A literature review also was conducted to learn what others recommend for handling such incidents. The survey results and published literature provide a basis for developing algorithms intended to guide surgeons in managing the contamination of a variety of autograft types used in plastic surgery.

## METHODS

**Figure 1:**
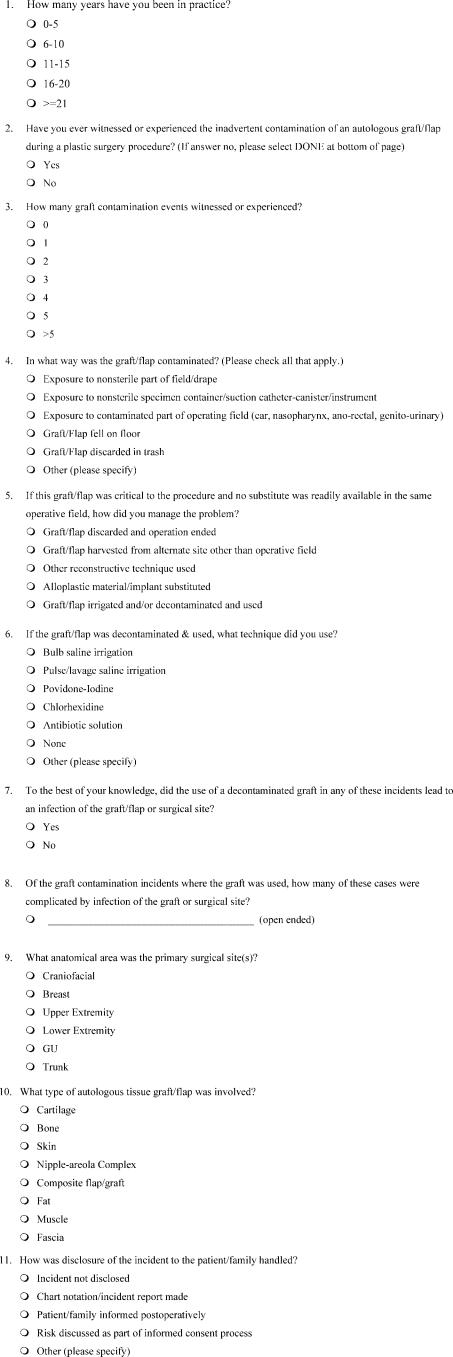
Survey sent to plastic surgeons on management of contaminated grafts.

We developed a questionnaire for which a Web link was sent via e-mail by the Aesthetic Surgery Education and Research Foundation to members of the American Society of Aesthetic Plastic Surgery (ASAPS). Members were asked to participate in the survey and assured that their responses would be anonymous. The Web link connected surgeons to an 11-question online survey, shown in Figure 1, that was hosted by Survey Monkey (www.surveymonkey.com), a data collection and analysis company. The questionnaire was designed to obtain information on autograft contamination frequency, treatment preferences, clinical outcomes, and patient disclosure.

In conjunction with the survey, PubMed was searched for published literature related to contamination of fat, skin, cartilage, composite tissue, and bone grafts, as well as proposed management strategies and appropriate decontamination techniques.

## RESULTS

Although 1900 ASAPS members were invited to participate in this anonymous online survey, only 223 surgeons (12%) submitted the questionnaire. The survey began by asking how long surgeons have been in practice. Of the 223 responders, 87% have been practicing for 10 or more years, with 65% in practice for more than 16 years.

Surgeons were then asked whether they had witnessed or experienced a graft contamination. The 30% of respondents who answered “no” were directed to stop and submit the survey. The 70% who said they had experienced a graft contamination represents at least 157 surgeons; however, there was a 3-surgeon inconsistency, with 160 answering questions that specifically asked about contamination experience. It seems that 3 surgeons who answered “no” on question 2 should have checked “yes” because they did, in fact, have graft contamination experience.

Reasons for the low (12%) response rate are unknown. Surgeons may not have had time to complete it or were not interested in the topic. Another possibility is that surgeons did not want to admit (even anonymously) that they have had a graft contamination incident. The fact that 87% of respondents have been in practice for at least 10 years may reflect a greater willingness to admit unintentional errors by more experienced surgeons. Because of the low response rate, the survey cannot be viewed as representative of all ASAPS members. Nevertheless, the results offer some interesting information about an issue not previously reported in the literature and rarely discussed openly by colleagues.

Among survey respondents, 70.4% (*n* = 157) reported either witnessing or experiencing graft contamination during a plastic surgery procedure. As shown in Table 1, 33% of surgeons reported 2 occurrences, and almost 26% experienced 4 or more contaminated graft incidents. The reporting of 2 instances by 52 surgeons represents 104 contaminations. If this mathematical exercise is continued and the “more than 5” response is assumed to be 6, then the total number of contaminated graft experiences is at least 426 among the 156 surgeons who answered this question.

Multiple responses were allowed for many survey questions to capture a fuller range of experience for each surgeon. The most frequent method of contamination, reported by 75% of respondents, was a graft or flap falling on the floor (Table 2). A distant second, at 44%, was exposure to a nonsterile part of the field/drape. All other reasons for contamination shown in Table 2 are equally accidental.

The predominant anatomical site undergoing surgery at the time of autograft contamination was the craniofacial region, at 66% (Table 3). This may be because craniofacial grafts tend to be very small and therefore may be more easily knocked off a table or thrown in the trash. Contamination also occurs in autografts of the lower extremity, breast, trunk, upper extremity, and genitourinary areas.

Table 4 shows the types of contaminated autografts. Skin grafts received the most responses (61%), followed by cartilage grafts at 39%. Nipple-areolar complex grafts and bone grafts were the only other types reported by more than 20% of respondents.

No surgeon abandoned a procedure after graft contamination. Instead, 94% decontaminated the graft and proceeded with their surgical plan (Table 5). Only 7% harvested another graft, 4% used another reconstructive technique, and 2% substituted alloplastic material. The methods of decontamination are shown in Table 6, with the favored techniques being the use of povidone-iodine (polyvinyl pyrrolidone or PVP-I) (54%) or antibiotic solution (50%). Responses to the “other” category for this question included the use of systemic antibiotics or washing the graft with other agents.

More than 98% of respondents who had contaminated graft experience said that its use did not, to their knowledge, lead to an infection. Three decontaminated grafts (1.9%) were associated with clinical infection, but this infection rate is comparable to that reported for clean noncontaminated surgical cases.^1^ One surgeon did not specify the location, 1 reported a systemic infection, and the third, a surgical site infection. Two of these surgeons had used PVP-I and 1 used bulb saline with antibiotic solution for decontamination.

Sixty percent of responding surgeons did not disclose the contamination incident to patients. Other actions taken are shown in Table 7, but only 21% said they had informed the patient/family postoperatively. The survey choice of “chart notation/incident report made” (selected by 25% of respondents) was poorly phrased because a chart notation is quite different from submitting an incident report to the surgical facility. One respondent noted that 1 incident was not disclosed because the possibility had been discussed in the informed consent process, but in another case the patient was told because the consent did not mention this possibility.

## DISCUSSION

Inadvertent contamination of autografts presents a dilemma for surgeons, yet the plastic surgery literature lacks specific discussions of clinical experience with the management or outcome of contaminated grafts. Although our search for relevant articles focused on autografts, bone and tendon allografts have been studied more than other types, with much of the literature appearing in orthopedic journals. Most of articles described either in vitro experiments or in vivo animal studies of sterilization agents used after intentional microbial inoculation. Extrapolating data from such experiments to clinical practice is difficult.

In our survey, the most common source of contamination resulted from a graft falling on the floor. Several investigations have cultured grafts intentionally dropped and left on an operating room (OR) floor for as little as 15 seconds. Although one study found no positive results of cultures on contaminated samples,^2^ others reported that between 58% and 96% of dropped grafts became contaminated.^3^–^5^ As proof of the risk to which patients may be exposed, 90% of rabbits receiving uncleansed contaminated grafts developed infections.^6^

The literature agrees that a dropped graft can be safely used if sterilized before placement. Among survey respondents, more than 94% did decontaminate and use the graft. Harvesting another graft is often not a practical option and/or can cause additional morbidity. Substitute alloplastic material may not be available, and obtaining an allograft can take days; furthermore, either of these approaches would require patient consent. Thus, abandoning a procedure with the idea of rescheduling later is impractical and probably not in the best interests of patients.

### Povidone-Iodine

The graft decontamination agent used by a majority of survey respondents (54%) was PVP-I. However, much of the literature suggests this readily available solution is not the best choice. Some comparison studies found PVP-I to be effective for bone contamination,^7^,^8^ yet one of these concluded that 10% PVP-I did not completely decontaminate femoral heads, and higher levels of contamination with *Staphylococcus epidermidis* required more than 10 minutes of soaking.^8^

The antimicrobial effectiveness of PVP-I has been challenged by Stanford and colleagues,^9^ who found that 10% PVP-I did not decontaminate cadaver patellar bone-tendon autografts even after 30 minutes of soaking or washing with agitation. Other studies comparing different antimicrobials and antiseptics determined that PVP-I was either ineffective or the least effective decontaminate tested.^5^,^10^

Some decontamination experiments have concluded that PVP-I and chlorhexidine gluconate (CGH [Hibiclens]) are toxic to bone cells, even at low concentrations of 1%.^11^ Although both agents reduced the number of bacterial colony counts, both also decreased the number of osteoclasts and impaired osteoblast function, as did bacitracin (Bacitracin) wash. Another study concluded that concentrations higher than 5% PVP-I were toxic to osteoblasts and intact tibiae at 2 minutes of exposure.^12^ In this study, however, bacitracin was not cytotoxic.

PVP-I also may damage fibroblasts. A 15-minute exposure to 10% PVP-I was found to kill 100% of human fibroblasts, and concentrations as low as 1% PVP-I were toxic.^13^ In this study, bacitracin was not toxic to fibroblasts. Balin and Pratt^14^ further demonstrated that an even weaker concentration of 0.1% PVP-I completely inhibited growth of adult skin fibroblasts. Fibroblast effects also were evident in human donor corneas decontaminated with concentrations higher than 0.5% PVP-I after a 2-minute soaking time.^15^ Moreover, higher PVP-I concentrations and longer soaking times were not completely effective in eliminating contaminates.

Because of its toxicity to osteoblasts and fibroblasts, as well as doubts about its antimicrobial effectiveness, PVP-I does not seem to be the best option for autograft decontamination. If it is used, a low concentration may be preferable.

### Antibiotic solutions

Antibiotic solution was the second most commonly used method of decontamination, reported by 50% of survey respondents. They were not asked to specify the antibiotic, but multiple solutions have been tested in investigational studies. The safety of antibiotics for irrigation and decontamination of various tissue types is widely accepted. As examples, bacitracin has been established as safe,^3^,^11^,^12^ as have rifampicin^4^ (Rifadin) and the combination of neomycin–polymyxin B.^5^ There is less agreement about effectiveness. Cephalosporins have been popular decontamination solutions,^16^ although their efficacy is not impressive and they are therefore not recommended by some investigators.^4^,^7^

Bhandari et al,^11^ who used 2-, 5-, and 10-minute soak times in bacitracin, found it to be the least effective of decontamination solutions they tested except for normal saline. An investigation that exposed patellar tendon allografts to an OR floor for 3 minutes and then soaked them in a bacitracin–Polymyxin B sulphate solution for 15 minutes determined that 30% of the grafts cultures' results were positive.^3^ The principal behind using a combination antibiotic solution is to broaden the spectrum of microbial coverage because grafts may be contaminated by a variety of organisms.

### Chlorhexidine gluconate

Several studies recommended CHG as the preferred decontamination agent. For example, an investigation of human anterior cruciate ligament grafts dropped on an OR floor for 15 seconds found that 4% CHG had the greatest sterilization impact, with only 2% (1 of 50) of the autografts remaining cultures' results were positive after a 90-second soak.^5^ This was compared to 6% for neomycin/Polymyxin B sulphate solution and 24% for PVP-I. In a study of harvested cadaveric skin grafts, there was a significantly diminished bacterial contamination rate (12% with PVP-I vs 2% with CHG) and the presence of fewer culture-positive species with CHG.^17^

An investigation by Goebel and colleagues^10^ inoculated rabbit patellar tendon-bone grafts with 2 different *Staphylococcus* species, then soaked the grafts for 30 minutes in 1 of 3 antimicrobial solutions, followed by a brief saline rinse.^10^ The gentamicin-clindamycin-polmyxin solution and the PVP-I were both 100% *ineffective* for decontaminating the grafts, but 4% CHG killed 100% of the *Staphylococcus* species. A second phase of this study determined that a 30-minute soak in 4% CHG successfully decontaminated grafts inoculated with *Pseudomonas aeruginosa*, *Escherichia coli*, *Staphylococcus aureus*, or *Enterococcus faecalis*. The triple antibiotic solution was more effective for decontaminating grafts exposed to *Klebsiella pneumoniae*.

Burd and colleagues^18^ confirmed Goebel's findings for tensor fascia lata and Achilles tendon-calcaneus grafts in humans. Low-power irrigation with 1 L or 3 L of a gentamicin-clindamycin-polmyxin solution did not decontaminate the grafts, nor did benzalkonium chloride or castile soap. Some cultures' results were positive after 1 L of irrigation with CHG, but 3 L of 4% CHG completely disinfected all tissues, even those inoculated with *K pneumoniae*. The experiment was repeated using a 2% CHG solution, which was equally effective.^18^ The use of power irrigation rather than a bath reduced the CHG decontamination time to less than 12 minutes (in comparison with 30 minutes in the Goebel study).

Although Burd's experiments determined that concentrations of CHG less than 2% were not effective, another investigation found that 0.05% CHG delivered by pressurized jet lavage for 1 minute followed by a 1-minute saline rinse successfully removed 99.8% of contaminating bacteria on rat cartilage without damaging its metabolic activity.^19^ The safety of CHG for cartilage also has been demonstrated through daily intraarticular injections of 4% CHG into rabbit knees for 5 days. After this lengthy exposure, no histologic changes were detected in the cartilage in comparison with noninjected controls.^16^

Only 1 surgeon who responded to the survey reported using CHG for a contaminated graft. Yet, the effective decontamination potential of CHG has been clearly demonstrated through in vitro and in vivo investigations of bone-tendon, ligament, cartilage, and skin grafts. Both PVP-I and CHG are readily available in operating rooms or easy to obtain quickly. Mixed-drug antibiotic solutions typically must be ordered from the pharmacy, which can lead to lengthy delays.

### Pulse lavage

High- and low-pressure pulse lavage (1–75 psi) have long been recognized as safe and effective for decontamination of soft tissues.^11^ However, bone may be prone to damage from lavage, especially high-pressure pulses. A trial involving rats with fresh, noncontaminated leg fractures found that fractures exposed to high-pressure lavage healed significantly more slowly than did those receiving bulb irrigation.^20^ Another study inoculated canine and human tibiae with *S aureaus* to study removal of adherent bacteria using sterile saline at high-pressure versus low-pressure lavage.^21^ Both high- and low-pressure lavage thoroughly decontaminated the bone, yet both also caused periosteal separation, and high-pressure lavage caused cortical fissures and structural defects in the bone.

Another investigation of canine cortical tibiae inoculated with *S aureus* found that the bacterial count was reduced significantly more when delivered with low-pressure pulse lavage for 2 minutes than when washed for 2 minutes.^11^ There was no significant difference between wash and lavage when 1% PVP-I and 1% CHG were tested. In addition, low-pressure pulse lavage with 3 L of sterile saline alone was determined to be more effective for decontaminating bone allografts exposed to an OR floor than was rinsing with either cefuroxime (Ceftin) or rifampicin.^4^

Not only does the literature suggest that high-pressure pulse lavage may be harmful for bone grafts; high-pressure lavage systems are not always readily available in ORs, nor do they seem a practical solution for decontaminating the sometimes very small grafts used in plastic surgery. The addition of low-pressure pulse lavage, however, seems to play an important role in graft decontamination.

## RECOMMENDATIONS

The literature contains many conflicting results about which antimicrobial solution is most effective, which concentration or volume of a solution is better, or how long grafts should be exposed to a solution. One point is clear that an autograft can be contaminated in the average OR within seconds. We must therefore assume that a clinical infection may result from graft contamination, and 3 survey respondents did report infections following contamination incidents. It is therefore incumbent upon the surgeon to deal with such incidents in an appropriate manner.

**Figure 2:**
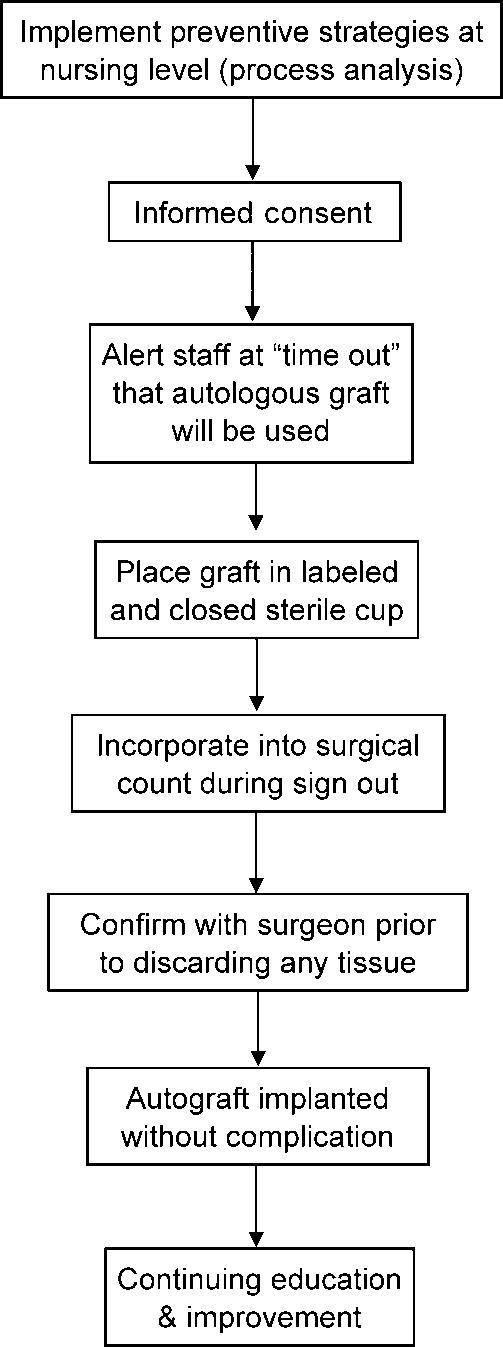
Perioperative autograft harvest algorithm.

Prevention should be the first priority. The algorithm shown in Figure 2 outlines perioperative steps for reducing the chance of graft contamination. The process analysis should include OR staff members to determine how graft contamination has occurred or might occur. All OR staff members should be explicitly alerted at the beginning of a case, during the mandatory “time-out” period, that an autologous graft will be used, and they should be aware of a graft's location at all times. To minimize the chances of contamination, place the graft in a labeled, sterile container with a closed lid, and place it on the widest table available and away from the table edge or instruments. In addition, limit handling and exchanges of a graft between OR personnel to reduce the chance of dropping or exposure to a nonsterile area. Include the graft in the surgical count during the checkout process when a surgical technician is relieved, and have the surgical assistant confirm with the surgeon before discarding any tissue.

The written and verbal informed consent process for a procedure that will employ a graft seeks permission to harvest. This provides an ideal opportunity to convey the potential for graft contamination so that patients will be aware, in advance, that accidents can happen.

**Figure 3:**
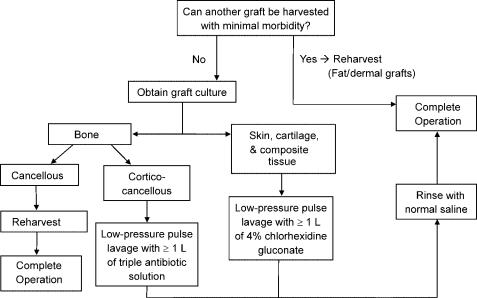
Intraoperative algorithm for contaminated autograft.

The Figure 3 algorithm presents the steps to take if an autograft does become contaminated. Before decontamination, culture the graft to find out what organisms might have contaminated it. Recently, experts have recommended culturing all grafts, including allografts, before implantation so that appropriate antibiotics can be prescribed should infection develop.^22^ Include *Clostridium* in the culture because it has been implicated in deaths of patients who received tissue bank allografts.^23^ In addition, the Centers for Disease Control and Prevention recommends all allografts be sterilized just before implantation.^24^

We located no studies on the decontamination of fat grafts, but the limited added morbidity from harvesting more fat precludes the need for decontamination. Skin, cartilage, and composite tissue should be sterilized with at least 1 L of 4% CHG, preferably with low-pressure pulse lavage. Several studies recommend 3 L of decontamination solution, but this large volume does not seem necessary for the very small grafts typically used in plastic surgery. Furthermore, the addition of low-pressure lavage should reduce the need for larger volumes and for prolonged soaking times. We recommend reharvesting cancellous bone, but cortico-cancellous bone may be decontaminated with low-pressure lavage and 1 L of triple antibiotic solution, the only option not found to be cytotoxic to bone. Regardless of the decontamination agent used, rinse the graft with normal saline as the final step.

**Figure 4:**
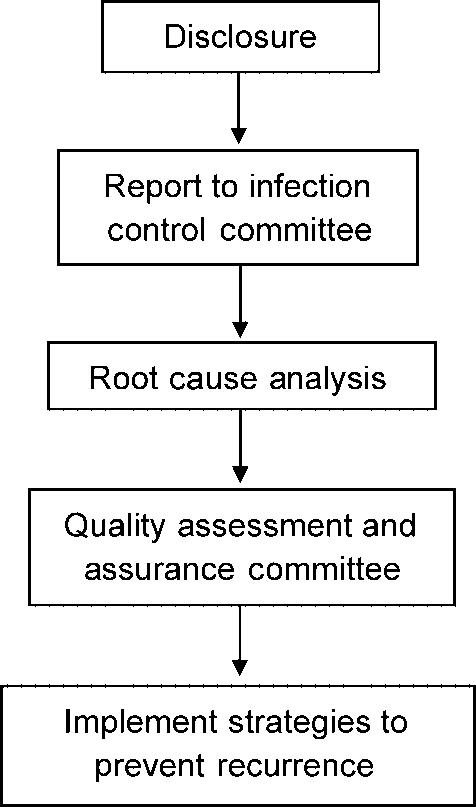
Postoperative algorithm for contaminated graft.

The Figure 4 algorithm outlines a process for postoperative management. We advocate full disclosure to the patient. If the informed consent process is handled properly, patients are aware of and prepared for the possibility of contamination. Failure to disclose the incident can increase the likelihood of a malpractice suit.^25^,^26^ A focus group of patients identified what they want to hear following a medical error: full disclosure; clinical impact; causative factors; methods of future error prevention; and emotional support, including an apology.^27^

Many patients cite their desire to prevent similar adverse events as justification for a malpractice claim,^28^,^29^ yet a discussion of ways to prevent future errors is not a routine part of disclosure.^30^ Therefore, root cause analysis along with an incident report should be part of postoperative process for preventing future incidents. Furthermore, engaging in the quality assurance review process is protected, privileged communication and non-discoverable in a legal proceeding as a way to encourage well-intentioned healthcare providers to report adverse events for quality improvement purposes.

## CONCLUSION

Typically, incidents of graft contamination are handled according to a surgeon's personal preference or a particular institution's policies. Because our survey results suggest that autologous graft contamination may not be as rare as we'd like to believe, this article proposes the graft decontamination procedures outlined in the presented algorithms. Perhaps, the most important point to emerge from our literature review is that PVP-I is not recommended for graft decontamination by many investigators. Disclosure, reporting, and privileged quality improvement should not be overlooked as part of the prevention process.

## Figures and Tables

**Table 1:** Number of graft contaminations reported by 156 surgeons with contamination experience

No. of incidents	No. of Surgeons (%)
1	43 (27.5)
2	52 (33.3)
3	21 (13.5)
4	11 (7.1)
5	2 (1.3)
>5	27 (17.3)

**Table 2:** Causes of graft contamination^a^

	Percentage of surgeons reporting this experience
Graft/flap fell on floor	75
Exposure to nonsterile part of field/drape	44.3
Exposure to contaminated part of operating field (ear, nasopharynx, anorectal, genitourinary)	28.7
Graft/flap discarded in trash	28.1
Exposure to nonsterile specimen container/suction catheter-canister/instrument	16.9
Other	1.9

^a^Multiple responses allowed. Data for 312 contamination incidents reported by 160 surgeons.

**Table 3:** Anatomical area where graft was to be placed^a^

Anatomical area	Response, % (no. of experiences)
Craniofacial	65.8 (104)
Lower extremity	27.2 (43)
Breast	25.3 (40)
Trunk	17.7 (28)
Upper extremity	17.1 (27)
Genitourinary	2.5 (4)

^a^Multiple responses allowed. Data for 246 contamination incidents reported by 158 surgeons.

**Table 4:** Contamination incidents by graft type^a^

Graft/flap type	Response, % (no. of experiences)
Skin	61.4 (97)
Cartilage	39.2 (62)
Nipple-areolar complex	22.2 (35)
Bone	21.5 (34)
Composite flap/graft	12.7 (20)
Muscle	11.4 (18)
Fascia	7.6 (12)
Fat	3.8 (6)

^a^Multiple responses allowed. Data for 284 contamination incidents reported by 158 surgeons.

**Table 5:** Management of contaminated graft^a^

	Response, % (no. of experiences)
Graft/flap irrigated/decontaminated and used	94.4 (151)
Graft/flap harvested from alternate site other than operative field	6.9 (11)
Other reconstructive technique used	3.8 (6)
Alloplastic material/implant substituted	1.9 (3)
Graft/flap discarded and operation ended	0

^a^*Multiple responses allowed. Data for 160 reporting surgeons.

**Table 6:** Decontamination technique employed if graft was used^a^

Method	Response, % (no. of experiences)
Povidone-Iodine	54.1 (85)
Antibiotic solution	50.3 (79)
Bulb saline irrigation	42.7 (67)
Pulse/lavage saline irrigation	11.5 (18)
Other (please specify)	3.8 (6)
None	3.2 (4)
Chlorhexidine gluconate	0.6 (1)

^a^Multiple responses allowed. Data for 260 contamination incidents reported by 157 surgeons.

**Table 7:** Contamination incident disclosure

	Response, % (no. of experiences)
Incident not disclosed	60 (96)
Chart notation/incident report made	25 (40)
Patient/family informed postoperatively	20.6 (33)
Risk discussed as part of informed consent	11.9 (19)
Other	3.8 (6)
^a^Multiple responses allowed. Data reported by 160 surgeons.	
